# Virologic and immunologic outcome of HAART in Human Immunodeficiency Virus (HIV)-1 infected patients with and without tuberculosis (TB) and latent TB infection (LTBI) in Addis Ababa, Ethiopia

**DOI:** 10.1186/1742-6405-10-18

**Published:** 2013-07-10

**Authors:** Desta Kassa, Gebremedhin Gebremichael, Yodit Alemayehu, Dawit Wolday, Tsehaynesh Messele, Debbie van Baarle

**Affiliations:** 1 Infectious and non-infectious diseases research directorate, Ethiopian Health and Nutrition Research Institute (EHNRI), P.O. Box 1242, Addis Ababa, Ethiopia; 2 Department of Internal Medicine and Infectious Diseases and Department of Immunology, University Medical Center Utrecht, Utrecht, The Netherlands; 3 Medical Biotech Laboratory, Addis Ababa, Ethiopia

**Keywords:** HIV, Tuberculosis, HAART

## Abstract

**Background:**

HIV/TB coinfection remains a major challenge even after the initiation of HAART. Little is known about *Mycobacterium tuberculosis (Mtb)* specific immune restoration in relation to immunologic and virologic outcomes after long-term HAART during co-infections with latent and active TB.

**Methods:**

A total of 232 adults, including 59 HIV patients with clinical TB (HIV + TB+), 125 HIV patients without clinical TB (HIV + TB-), 13 HIV negative active TB patients (HIV-TB+), and 10 HIV negative Tuberculin Skin TST positive (HIV-TST+), and 25 HIV-TST- individuals were recruited. HAART was initiated in 113 HIV + patients (28 TB + and 85 TB-), and anti-TB treatment for all TB cases. CD4+ T-cell count, HIV RNA load, and IFN-γ responses to ESAT-6/CFP-10 were measured at baseline, 6 months (M6), 18 months (M18) and 24 months (M24) after HAART initiation.

**Results:**

The majority of HIV + TB- (70%, 81%, 84%) as well as HIV + TB + patients (60%, 77%, 80%) had virologic success (HIV RNA < 50 copies/ml) by M6, M18 and M24, respectively. HAART also significantly increased CD4+ T-cell counts at 2 years in HIV + TB + (from 110.3 to 289.9 cells/μl), HIV + TB- patients (197.8 to 332.3 cells/μl), HIV + TST- (199 to 347 cells/μl) and HIV + TST + individuals (195 to 319 cells/μl). Overall, there was no significant difference in the percentage of patients that achieved virologic success and in total CD4+ counts increased between HIV patients with and without TB or LTBI. The *Mtb* specific IFN-γ response at baseline was significantly lower in HIV + TB + (3.6 pg/ml) compared to HIV-TB + patients (34.4 pg/ml) and HIV + TST + (46.3 pg/ml) individuals; and in HIV-TB + patients compared to HIV-TST + individuals (491.2 pg/ml). By M18 on HAART, the IFN-γ response remained impaired in HIV + TB + patients (18.1 pg/ml) while it normalized in HIV + TST + individuals (from 46.3 to 414.2 pg/ml).

**Conclusions:**

Our data show that clinical and latent TB infections do not influence virologic and immunologic outcomes of ART in HIV patients. Despite this, HAART was unable to restore optimal TB responsiveness as measured by *Mtb* specific IFN-γ response in HIV/TB patients. Improvement of *Mtb*-specific immune restoration should be the focus of future therapeutic strategies.

## Background

Human immunodeficiency virus/Acquired immunodeficiency syndrome (HIV/AIDS) associated morbidity and mortality has reduced substantially since the introduction of Highly Active Antiretroviral Therapy (HAART) in the mid 90’s [1,2]. Access to Antiretroviral Therapy (ART) in low and middle income countries has been expanded following the launche of “3 by 5” global initiative [3], though only 54% of those eligible for ART were on treatment by 2011 [2]. In Ethiopia, where free ART was started in 2005, >250,000 (~79%) of the adults requiring ART were actually treated [4].

The primary goal of HAART is to suppress HIV-1 RNA lower than the detection level (LDL) of the assay within 3 to 6 months on treatment and restore immunologic function, to reduce morbidity and mortality, to reduce vertical transmission, and improve quality of life [5]. However, there are still un-resolved problems including early mortality [6], incomplete responses [7], variations in HAART outcomes [8], lack of universal consensus to define treatment failures and time to start ART [9], drug resistance [10] and lost to follow-ups [7].

While HIV RNA testing is the golden standard to monitor patients on ART [8], due to costs and technical demands of the HIV RNA test, CD4+ T cell measurements are recommended for resource poor settings [11]. Immunologic parameters, however, have lower performance to identify virologic failures which could lead to premature change or to continuous use of failed regimens reviewed in [12]. This leads to higher morbidity and mortality rates and more complex resistance in settings where virologic tests are not available [13]. Therefore, accurate diagnosis of treatment failure is necessary in settings where free ART service is accelerating and patient monitoring is exclusively dependent on clinical and CD4+ T cell measurements like in Ethiopia.

Furthermore, despite that HAART has significantly reduced morbidity and mortality in HIV/TB patients [14], studies showed defects of immune response in HIV/TB patients on HAART including suboptimal restoration of CD4+ T cells in number, phenotype and function [15,16], and incomplete TB specific immune restoration [17]. Higher TB incidence in individuals on continuous HAART as compared to the HIV negative local population, which could be due to incomplete immune restoration specific to TB, was also reported [18]. However, data regarding the magnitude of immune restoration specific to *Mtb* in relation to CD4+ and virologic responses to long-term HAART in patients with TB and LTBI is limited.

Overall, although extensive studies aiming to evaluate ART outcomes have been performed, the studies are predominantly from developed countries, and they are different in study design, ex-/inclusion criteria, ethnicity, ART experience, ARV regimen, duration, and definitions, which makes it difficult to generalize HAART outcomes in different countries [19]. Especially in Ethiopia, like in many HIV/TB endemic settings, little data is available [20,21] regarding HAART outcome in patients with and without active TB and LTBI.

In summary, these studies strongly support the need of recent data from well defined longitudinal cohort studies on HAART, which is crucial to provide answers and insights to the HAART related challenges and develop and update national ART guidelines [19,22].

The aim of this observational cohort study which comprised three clinical groups including HIV + TB+, HIV + TB-, and HIV-TB + patients, and two control groups including HIV-TST+, and HIV-TST- individuals , was to evaluate the long-term outcome of HAART by comprehensively measuring key parameters including mortality, virologic and immunologic responses, and *Mtb* specific immune restoration by measuring IFN-γ production in response to Early Secretory Antigenic Target-6/Culture Filtrate Protein-10 (ESAT-6/CFP-10).

## Results

### Characteristics of the study population at enrolment

Demographic, clinical and laboratory data of the study populations at baseline are shown in Table 1. A total of 232 participants were included. Thirteen HIV-TB+, 59 HIV + TB+, 125 HIV + TB- (of whom 43 were HIV + TST+, and 82 HIV + TST-), 10 HIV-TST+, and 25 Controls (HIV-TST-) were enrolled. HIV + TB + patients had lower numbers of CD4+ T cells (p = 0.003), total Lymphocyte count (TLC) (p = 0.001), Hemoglobin (Hgb) (g/dl) (p = 0.02), Body Mass Index (BMI) (Kg/m^2^) (p = 0.002), CD4% (p = 0.006), but higher HIV RNA levels (p = 0.02) than HIV + TB- patients. There was no significant difference in CD4+, TLC, Hgb, BMI, CD4% and HIV RNA tests between HIV + TST- and HIV + TST + individuals (data not shown). Of special interest, 10 (6.3%) of the ART naïve HIV patients had HIV RNA < 50 copies/ml (LDL) at enrolment. The mean CD4+ T cell count of these subjects was 476.1 (SD ± 260.7 cells/μl).

**Table 1:** Baseline characteristics of the study populations (n = 232)

	**HIV + TB+**	**HIV + TB-**	**HIV-TB+**	**HIV-TST+**	**HIV-TST**
	**(n = 59)**	**(n = 125)**	**(n = 13)**	**(n = 10)**	**(n = 25)**
**Demographic data**					
Age, years	33.1 ± 8.7	33.2 ± 7.3	28.5 ± 9.5 ^b^	26.6 ± 7.4,	24.6 ± 6.6
Female, n (%)	28 (47.5)	83 (66.4)	6 (46.2)	6 (60)	16 (64)
**Clinical data**					
CHBV coinfection, n (%)	11/57 (19.3)	8/112 (7.2)	3 (15)	2 (20)	1 (4)
WHO stage, n (%)					
I + II	3/50 (6)	80 (64)			
III + IV	47/50 (94)	45 (36)	NA	NA	NA
BMI, kg/m2	18.9 ± 3.1^a^	21.3 ± 3.5	18.9 ± 2.8	21.4 ± 2.5	21.4 ± 2.0
BMI < 18.50 kg/m^2^, n (%)	29 (49.2)	18/124 (14.6)	6 (46)	8 (80)	24 (96)
**Laboratory data**					
Hgb, g/dl	12.2 ± 3.7 ^a^	13.2 ± 2.5	13.5 ± 2.5 ^b^	16.4 ±1.8 ^c^	18.0 ± 2.1
Hgb < 12 g/dl (anemic), n (%)	23/43 (53.5)	29/107 (27.1)	2 (23)	0	0
CD4+ count/μl	173.7 ± 180.8 ^a^	279.2 ± 212.4	478.1 ± 253 ^b^	787.3 ± 274	754.8 ± 241
CD4+ category, n (%)					
< 100	27 (48.2)	26 (22.2)	1 (1.7)	0	0
100 –200	12 (21.4)	26 (22.2)	6 (10.0)	0	0
> = 201	17 (30.3)	65 (55.6)	53 (88.3)	10 (100)	25 (100)
TLC	1175 ± 754 ^a^	1626 ± 750	1485 ± 895 ^b^	1975 ± 1483	1.668 ± 559
HIV RNA (log10copies/ml)	4.5 ± 0.8 ^a^	4.1 ± 0.9	NA	NA	NA
HIV RNA category, n (%)					
LDL	1 (2.2)	9 (8.0)			
<= 100000	14 (30.4)	97 (85.8)			
>100000	32 (69.6)	16 (14.2)	NA	NA	NA

Data are means ± standard deviations (SD) unless stated; *n (%)* Number of patients, **CHBV** chronic Hepatitis B Virus, defined as the presence of hepatitis surface antigen (HBsAg) in the plasma, **BMI** Body mass index, **Hgb** Hemoglobin, **TLC** total lymphocyte, **HIV RNA** plasma viral load, **LDL** lower than detection limit (HIV RNA < 50 copies/ml), **WHO** World Health Organization, **NA**: not applicable.

^a^ Comparing HIV + TB + and HIV + TB-; *P < 0.05.*

^b^ Comparing HIV + TB + and HIV-TB+; *P < 0.05.*

^c^ Comparing HIV-TST + and HIV-TST- ; *P < 0.05.*

At enrolment, more than 50% of the HIV + TB + patients had advanced diseases stages (anemic, malnourished, WHO stages 3 plus 4, and immunosuppressed) while only 15-36% of the HIV + TB- patients had these advanced disease stages. There was also a higher percentage of chronic hepatitis B virus coinfection in the HIV + TB + patients, (19.3%), followed by the HIV + TB-, (7.2%), and healthy controls (4.2%) (Table 1).

### Outcome of HAART

#### ART initiation, mortality and follow-up status

A total of 113 (61.4%) HIV patients including 28 HIV + TB + and 85 HIV + TB- [of whom 31 were HIV + TST + and 54 were HIV + TST-], initiated ART and were followed for a median of 23.9 (IQR 22.5-24.4) months. The majority (78%) of the HIV + TB- patients received D4T + 3TC + NVP HAART regimens. At ART initiation, HIV + TB + patients had lower mean CD4+ T cells (*p = 0.005)*, CD4% (*p = 0.03)*, TLC (p = 008)*,* and BMI (*p < 0.0001)* than HIV + TB- patients (Table 2).

**Table 2:** Therapeutic, clinical and immunovirologic characteristic of the study participants at ART initiation (n = 113)

	**HIV + TB+**	**HIV + TB-**	***p value***
No of patients initiated ART	28	85	
**Demographic**			
Female, n (%)	14 (50)	56 (65.7)	
Age, years	34.1 ± 8.3	34.4 ± 7.7	
**Treatment**			
Mean follow-up days before ART initiation	78.7	37.7	
Mean delay in commencing HAART following TB treatment, days	100		
HAART regimen at ART initiation, n (%)			
D4T/3TC/NVP	4 (15)	66 (78)	
D4T/3TC/EVZ	11(41)	2 (2)	
AZT/3TC/NVP	7(26)	12 (14)	
AZT/3TC/EVZ	5 (16)	4 (5)	
Others		1 (1)	
Cortimoxzole treatment, n (%)	15 (83.3%)	58 (59.2%)	
**BMI and Laboratory values at ART initiation**			
BMI (kg/m^2^)	18.1 ± 2.5	21.3 ± 3.7	0.0001
CD4+ (cells/μl)	110.3 ± 71.3	197.8 ± 153.2	0.005
CD4%	10.1 ± 5.3	14.6 ± 9.9	0.033
TLC (cells/μl)	1078 ± 512	1521 ± 692	0.007
Hgb (g/dl)	13.4 ± 3.4	13.0 ± 2.5	0.68
HIV RNA (log_10_copies/ml)	4.3 ± 0.9	4.3 ± 0.9	0.73
**Follow-up outcomes**			
Deaths, n (%)	8/59 (13.6)	10/125 (8.0%)	

Data in mean ± standard deviation (SD) unless stated; *n* number of patients, D4T Stavudine, 3TC lamividine, NVP Nevirapine, EVZ Efavirenz, AZT Zidovudin, BMI Body mass index, Hgb Hemoglobin, TLC total lymphocyte count; M6, M18 and M24 = 6, 18 and 24 months after HAART initiation.

At 2 years, 18 (9.8%) patients died of which 63% within the first 24 weeks. The risk factors for early mortality in the HIV patients with and without TB were advanced disease stages including baseline CD4+ count <100 cells/μl (OR: 5.7, 95% CI 1.8 – 17.2, *p = 0.03*), WHO-stages III plus IV (OR: 4.8 95% CI 1.3-17.5, *p = 0.01*), and BMI < 18.5 kg/m2 (OR: 1.2 95% CI 0.37-3.8, *p = 0.77*) (data not shown).

Overall, at M6, M18 and M24 of follow-up time, 21/59 (35.6%), 21/51 (41.2%) and 23/41 (56.1) of the HIV + TB + patients; and 27/125 (21.6%), 36/120 (30%) and 43/115 (37.4%) of the HIV + TB- patients, respectively, were lost to follow-up (more than 60 days late for a scheduled date of clinic visit).

#### Virologic outcomes

**Figure 1:**
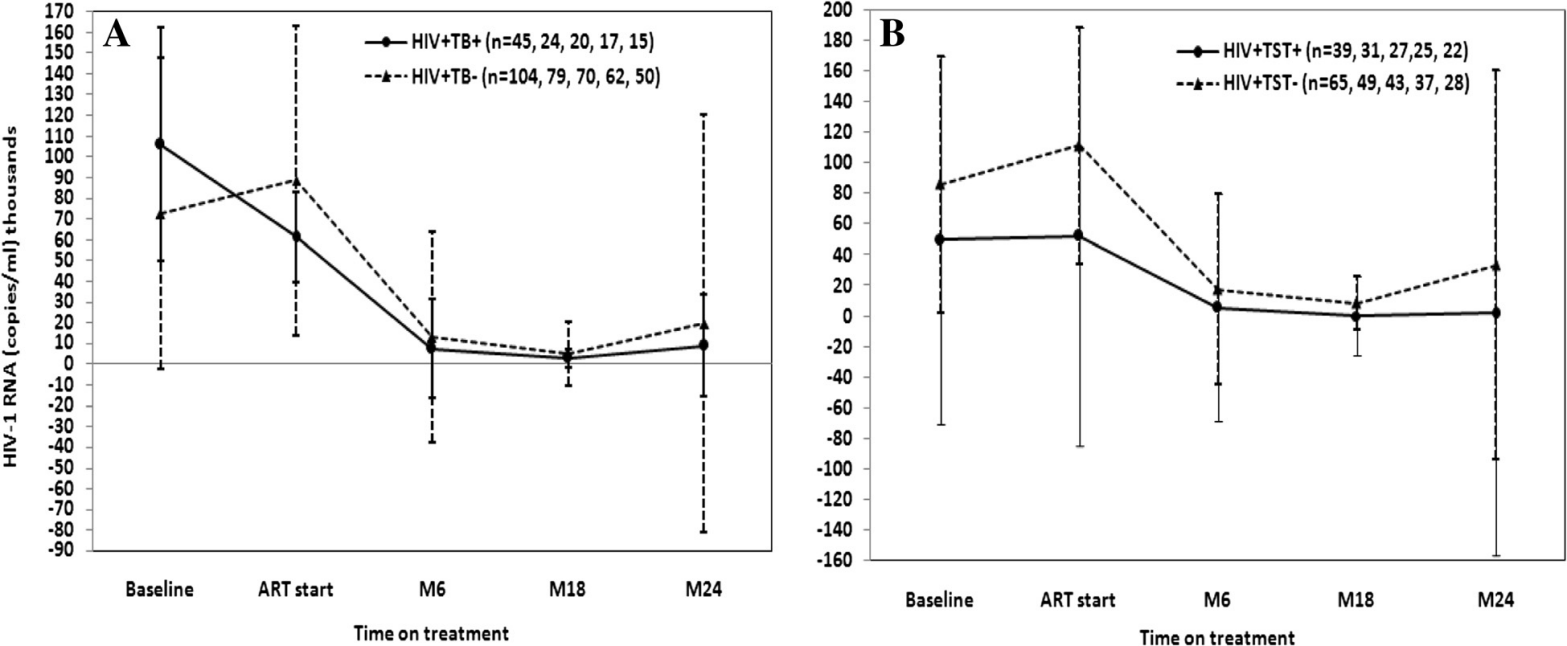
**Changes in HIV RNA level (copies/ml) over time after the start of HAART.** HIV RNA was measured at baseline, at ART start and at six month (M6), M18 and M24 of HAART in HIV patients with TB (HIV + TB+) (continuous line) and without TB (HIV + TB-) (dotted line) **(A)**, and HIV + TB- patients sub-grouped as those with TST positive (HIV + TST+) (continuous line) and TST negative (HIV + TST-) (dotted line) **(B)**. Values are shown as mean and standard deviation. n = number of participants per visit.

We measured virologic outcomes by determining the HIV RNA level as well as the proportion of patients with virologic success. In both HIV + TB + and HIV + TB- patients, the mean HIV RNA level significantly declined after HAART (p < 0.05 and p < 0.001, respectively) (Figure 1A). There was no significant difference in mean HIV RNA decline between patients with and without TB or LTBI (Figure 1A & B).

**Figure 2:**
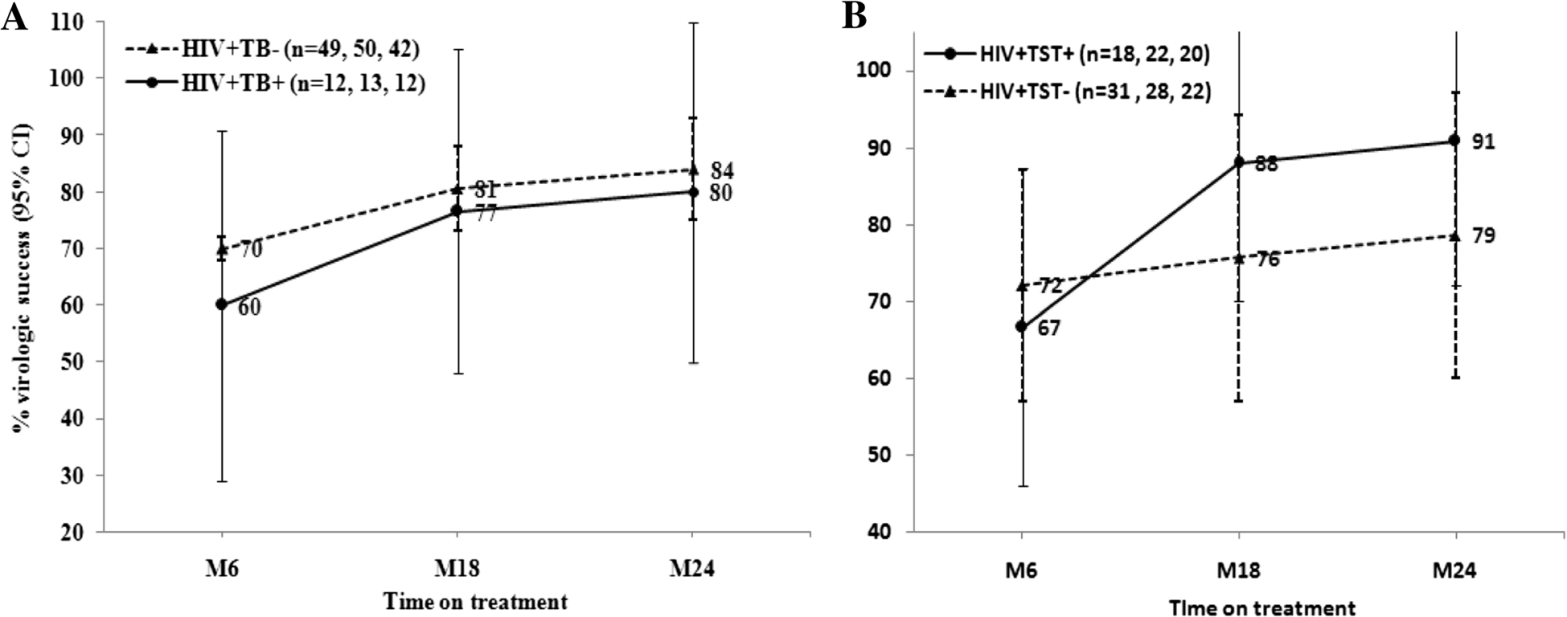
**Percentages of patients with virologic success (plasma HIV RNA < 50 copies/ml) with 95% confidence interval (95% CI) overtime on HAART.** HIV RNA was measured at six month (M6), M18 and M24 of treatment in HIV patients with TB (HIV + TB+) (continuous line) and without TB (HIV + TB- (dotted line) **(A)**, and HIV + TB- patients sub-grouped as those with TST positive (HIV + TST+) (continuous line) and TST negative (HIV + TST-) (dotted line) **(B)**. Percentage of patients with virologic success per follow up visit are shown on the line graphs; n = number of participants per visit.

By M24 on HAART, the majority of the individuals (>80%) achieved virologic success (HIV RNA < 50 copies/ml). There was no significant difference in the proportion of patients achieving virologic success between individuals with and without TB or LTBI (Figure 2A & B). Moreover, whereas 28 (85%) of the patients on HAART maintained HIV RNA < 50 copies/ml at M6, M18 and M24 (ever suppressed), 5 (15%) never reached HIV RNA < 50 copies/ml (never suppressed)*.*

At M6, 13 (19%) HIV + TB- patients had virologic failure (HIV RNA >5000 copies/ml), for which baseline BMI < 18.5 kg/m^2^, CD4+ count < 100 cells/μl, and WHO stages 3 and 4 [OR: 3.8, 2.3, 4.9; *p* = 0.05, 0.32, 0.02, respectively] were risk factors. Other factors like sex (OR: 0.51) age (OR: 0.41), Hgb (OR: 0.19), and HIV RNA (OR: 0.89) were not associated with the risk of virologic failures (Table 3).

**Table 3:** Logistic regression analysis showing odds ratio for factors associated with the risk of immunologic and virologic failures of the HIV patients with no TB (HIV + TB-) at six months of ART

	**Risk of immunologic failure (an increase of CD4+ < 50 cells/μl) at 6 months (n = 56)**			**Risk of virologic failure (HIV RNA > 5000 cells/μl) at 6 months (n = 69)**		
	**OR**	**95% CI**	**P value**	**OR**	**95% CI**	**P value**
Sex						
Male	1			1		
Female	0.62	1.0-1.9	0.40	0.51	0.12-2.1	0.93
Age (years)						
36-60	1			1		
16–35	0.65	0.21-2.0	0.44	0.41	0.10- 1.7	0.21
BMI						
<18.50	1			1		
> = 18.50	0.90	0.25- 3.29	0.87	3.75	1.0-14.4	0.05
Hgb						
< 12	1			1		
> = 12	0.38	0.08-1.7	0.21	0.19	0.02-1.6	0.13
CD4+ count/μl						
> = 100	1			1		
<100	5.6	1.6-20.1	0.008	2.3	0.45- 11.2	0.32
> = 200	1			1		
>200	0.22	0.07-0.73	0.01	0.89	0.24-3.2	0.85
HIV RNA copies/ml						
> = 100000	1			1		
<100000	1.2	0.3- 5.3	0.82	0.38	0.04- 3.3	0.4
WHO stages						
I + II	1			1		
III + IV	4.3	1.4-13.4	0.01	4.9	1.2-19.9	0.02
TB coinfection						
No	1			1		
Yes	1.4	0.4-1.8	0.5	0.23	0.03-1.9	0.17

OR Odds ratio, 95% CI 95% confidence Interval; n = total number of participants who have CD4+ count and plasma HIV RNA measurement at 6 month of HAART.

#### Immunologic responses

**Figure 3:**
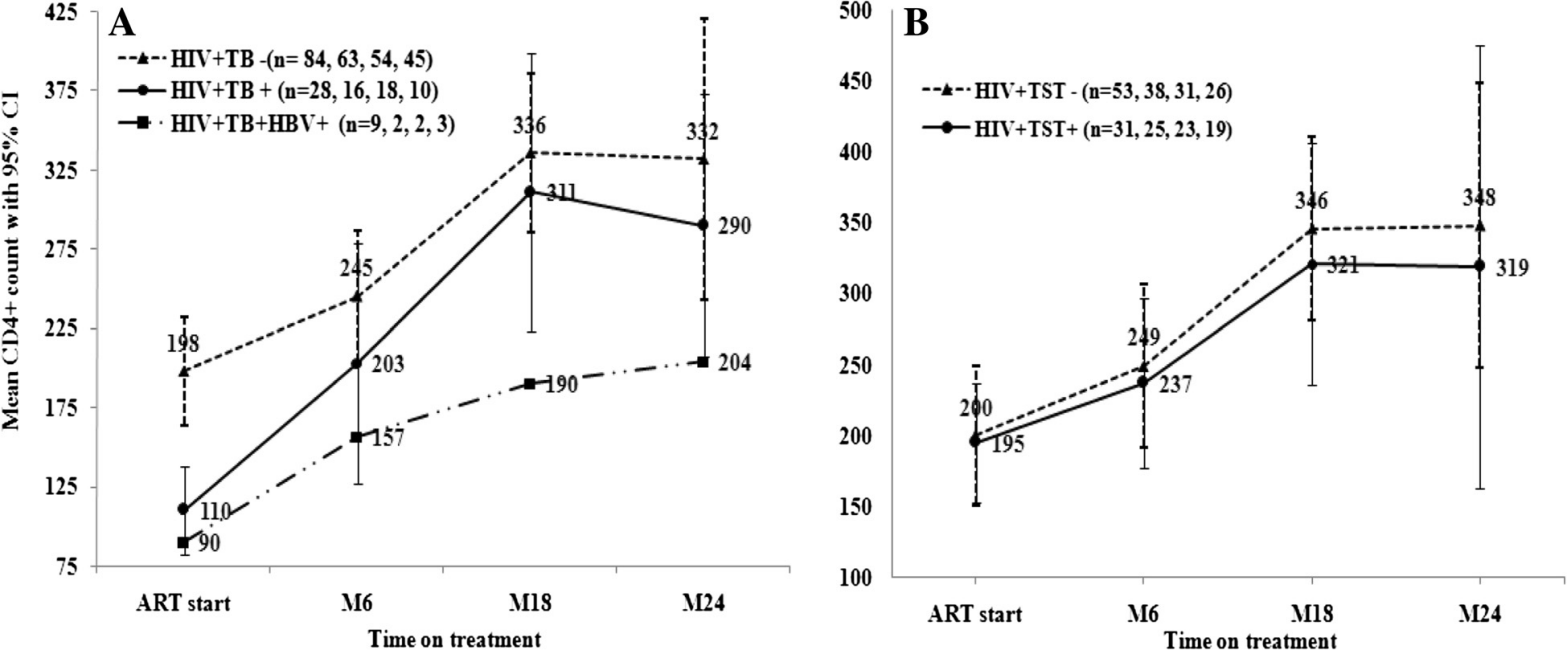
**Mean CD4+ count increase (cells/μl) with 95% Confidence interval (95% CI) over time after the start of HAART.** CD4+ T cell count was measured at ART start, six month (M6), M18, and M24 of HAART in HIV patients with TB (HIV + TB+) (continuous line), with no TB (HIV + TB-) (dotted line), and HIV patients with TB and HBV (HIV + TB + HBV) (dash line) **(A)**, and HIV + TB- patients sub-grouped as those with TST positive (HIV + TST+) (continuous line) and those TST negative (HIV + TST-) (dash line) **(B)**. Number of CD4+ T cell counts per follow-up visit are shown on the line graphs; n = number of participants per visit.

Quantitative restoration of CD4+ cells is one of the principal evidences for immune recovery during HAART. There was a significant increase in CD4+ T cell count at M6, M18 and M24 of HAART in both the HIV + TB- (*P <* 0.001 for all) as well as the HIV + TB + patients (p = 0.02, 0.001, 0.001, respectively); and in the HIV + TST + (*p =* 0.03, 003, 0.04, respectively) as well as in the HIV + TST- (*P <* 0.001 for all) (Figure 3A & B).

There was no significant difference in the total CD4+ T cells reached by M6, M18 and M24 in HIV + TB + vs. HIV + TB- (*p =* 0.37, 0.63 and 0.56, respectively) and in HIV + TST + vs. HIV + TST- (*p = 0.77, 0.62, and 0.74,* respectively) (Figure 3A & B). Interestingly, although the study population was small, HIV/TB patients coinfected with HBV (n = 9) showed the least increase in CD4+ T cells (Figure 3A).

There was an overall increase in CD4+ count over the two years with an average increase of 6.7 cells/μl per month in the HIV + TB- patients, and 5.9 cells/μl per month in the HIV + TB+. CD4+ increase was highest in the first six months. Despite lower CD4+ T cell count at ART initiation in HIV + TB + patients (110 CD4+ cell/μl) than HIV + TB- patients (198 CD4+ cell/μl) (p = 0.001), there was no significant difference in the net increase of CD4+ T cells per month as well as in the total CD4+ cells achieved at each time point between the patients with and without TB.

Overall, at two years on HAART, the total CD4+ cells in all clinical groups was still lower by more than two fold compared to healthy controls (HIV-TST-) (771 CD4+ cells/μl) (Figure 3A and B). Moreover, the proportion of patients that attained the critical CD4+ count of ≥ 200 cells/μl by two years on ART was 19 (68%) for HIV + TB-, and 8 (73%) for the HIV + TB+. Six (21%) of the HIV + TB- but none of the HIV + TB + patients had CD4+ count >500 cells/μl (super responders).

At M6, M18 and M24 of HAART, 45%, 30%, 42% of the HIV + TB- and 54%, 43%, and 30% of the HIV + TB + patients, respectively, were diagnosed with immunologic failure (an increase of less than 50 cells/ μl by M6, and less than 100 cells/ μl by M18 and M24 of HAART). The risk factors for immunologic failure at M6 of HAART for the HIV + TB- patients were WHO stages 3 + 4 [OR: 4.3, p = 0.01], and CD4+ < 100 cells/μl) [OR: 5.6, p = 0.008] at baseline (Table 3).

**Figure 4:**
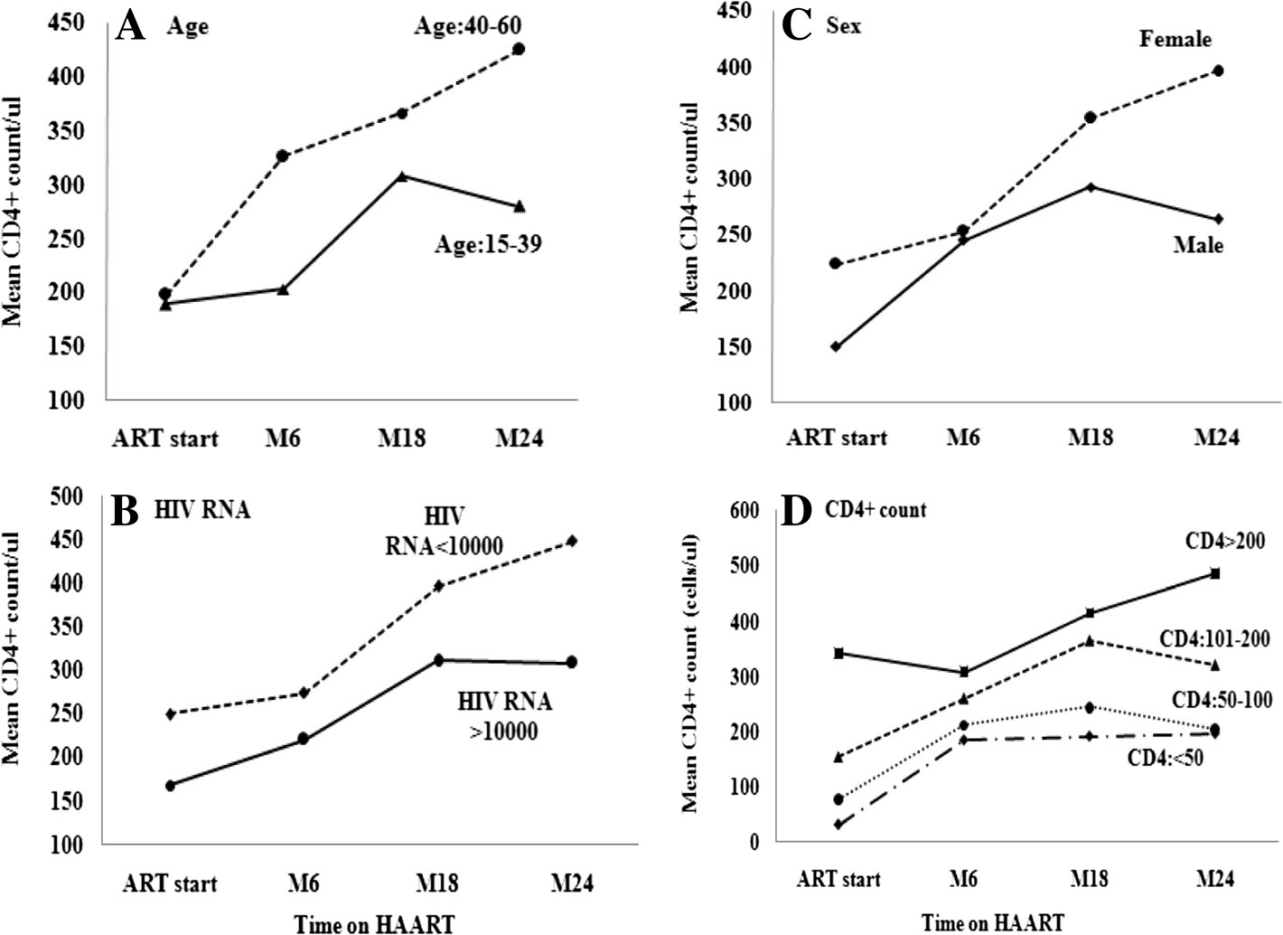
Mean CD4+ count increase (cells/μl) over time after the start of HAART in HIV patients with no TB (HIV + TB-) according to the baseline category of (A) Age, (B) HIV RNA (copies/ml), (C) Sex, and (D) CD4+ count (cells/μl).

As shown in Figure 4, for the HIV + TB- patients on ART, the CD4+ increase was steeper in women, in patients with older age, patients with baseline HIV RNA < 10000 copies/ml, and CD4+ count > 200 cells/μl with the difference becoming larger over time. More interestingly, the total CD4+ T cell count achieved in the HIV + TB- patients started ART at CD4+ > 200, was two fold higher than those started ART at < 200 cells/μl (495.0 vs. 243.2 cells/μl, respectively, (*P = 0.007*). This indicated that advanced pre-treatment immunodeficiency is the most important factor for diminished restoration of CD4 cell counts after HAART.

#### Mtb antigen specific IFN-γ response before and after HAART

**Figure 5:**
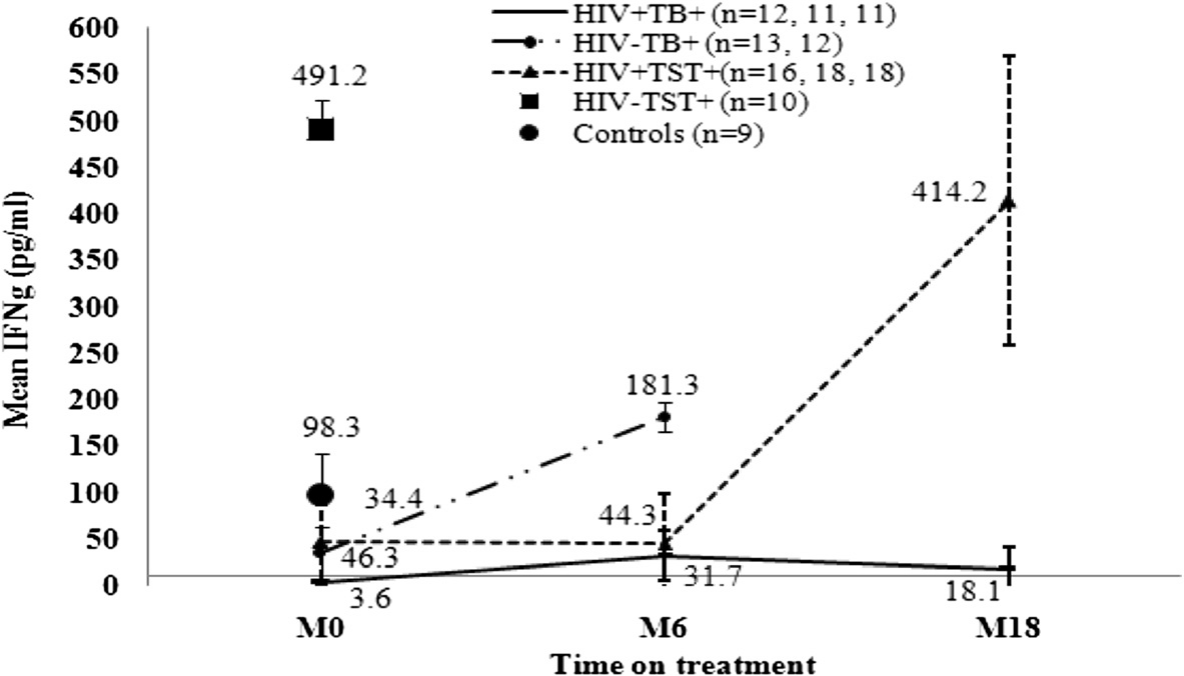
**Restoration of *Mtb *specific IFN-γ (pg/ml) response overtime after the start of HAART and TB treatment.** Level of IFN-γ (pg/ml) was measured at baseline (M0), and at six (M6) and M18 of treatment in 7th day culture supernatants of whole blood stimulated with *Mtb* specific (ESAT-6/CFP-10) antigen. **HIV + TB+**: HIV patients with TB (continuous line); **HIV-TB**: HIV negative TB patients (long-dash line); **HIV + TST+**: HIV positive tuberculin skin test (TST) positive patients (dot line); **HIV-TST+**: HIV negative TST positive individuals (square dot); and Controls (HIV-TST-) (circle dot). Mean of IFN-γ (pg/ml) with standard deviation per follow-up visit are shown on the line graph; n = number of participants per visit shown on the legend.

Although quantitative measurement of CD4+ counts and HIV RNA level provides a general insight in immune recovery, measuring qualitative restoration of TB specific immune responses will provide insight whether antigen specific immune responses are also restored. Therefore, we measured *Mtb* antigen (ESAT-6/CFP-10) specific IFN-γ responses during HAART (Figure 5). Compared to IFN-γ response in LTBI individuals (HIV-TST+) (491.2 pg/ml) at baseline, there was significantly lower IFN-γ production in HIV + TB + (3.6 pg/ml) (*p = 0.004*), in HIV-TB + (34.4 pg/ml) (*p = 0.004*), and in HIV + TST + (46.3 pg/ml) patients (*p = 0.002*). Moreover, IFN-γ production at baseline was significantly lower in HIV + TB + compared to HIV-TB + (*p = 0.02*) and HIV + TST + (*p = 0.04*) patients, and in HIV + TST + compared to HIV-TB + (*p = 0.004*).

In the HIV + TST + individuals, following initiation of HAART, although the level of IFN-γ did not change by M6 (44.3 pg/ml), it increased sharply after that and normalized by M18 of treatment (414.2 pg/ml) (*p = 0.007*) and reached similar level as HIV-TST + individuals (491.2 pg/ml) (p > 0.05). In contrast, there was no significant increase in *Mtb* specific immunity for the HIV patients with TB (HIV + TB+) on TB treatment and on HAART by M6 (31.7 pg/ml) as well as by M18 (18.1 pg/ml) of treatment. For the HIV negative TB patients (HIV-TB+) on TB treatment, mean IFN-γ production significantly increased by M6 (181 pg/ml) (p = 0.005)

**Figure 6:**
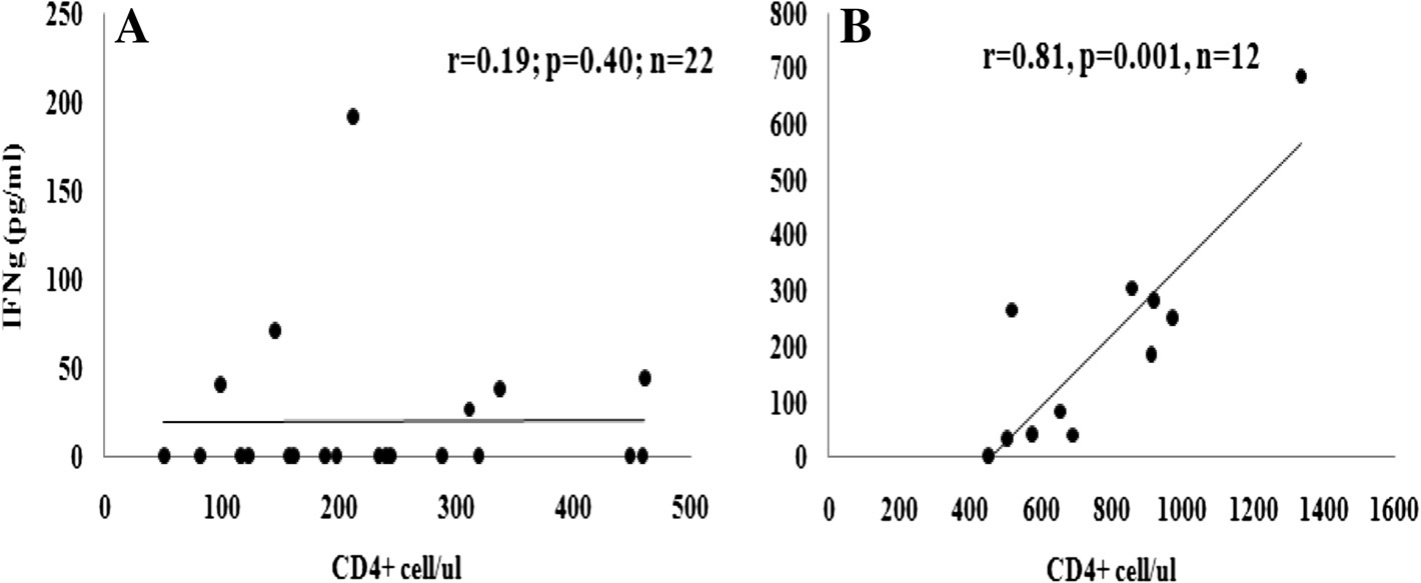
**Spearman correlation between IFN-γ production and absolute CD4+ count recovery after the start of HAART and TB treatment. (A)** HIV positive patients with active TB (HIV + TB+) at 6th and 18th months of HAART and TB treatment, and **(B)** HIV negative TB patients (HIV-TB+) at six months of TB treatment. IFN-γ secretion was measured in 7th day culture supernatants of whole blood stimulated with *Mtb* specific antigen (ESAT-6/CFP-10). r = correlation coefficient.

To see whether the poor *Mtb*-specific recovery is due to lower CD4 recovery, we correlated the increase in CD4+ cell count in the HIV + TB + patients after HAART with IFN-γ production upon stimulation with ESAT-6/CFP-10 (immune function specific to TB). No significant correlation between CD4+ T cell count recovery and IFN-γ production was found *(r = 0.19, p = 0.40, n = 22)*. In contrast, however, there was a strong positive correlation between the recovery of CD4+ cells and IFN-γ production in the HIV negative active TB patients (HIV-TB+) following TB treatment for six months *(r = 0.71, p = 0.0007, n = 19)* (Figure 6).

## Discussions

In this study, we determined the long-term outcome of HAART in HIV patients with and without TB and LTBI by comprehensively measuring HIV RNA suppression, CD4+ T-cell recovery, and immune reconstitution specific to *Mtb*.

The goal of ART is to suppress HIV-1 RNA below the detection limit of the assay within 12–24 weeks [5], or to less than 0 · 5-0 · 75 log copies/ml by 4 weeks [23]. In this study, 84% of the non-TB patients on HAART had HIV RNA <50 copies/ml at 24 months on ART. This is comparable to most studies from Africa [24,25], Europe [15,26], and United States [27].

Nevertheless, 19% of the non-TB patients in this study were virologic failures (HIV RNA > 5000 copies/ml) at 6 months of ART. Reports from Cameroon [28] and Brazil [29] also showed virologic failure (HIV RNA > 400 copies/ml) in 13% and 28% of patients at 6 months on ART. As reported by Tuboi SH *et al.*[29], malnutrition and advanced WHO stages were risk factors for virologic failure in this study, which strongly indicate the need of earlier identification of eligible patients and earlier initiation of HAART for better treatment outcome.

Interestingly, comparable to other reports [15,30], also 80% of the HIV patients with TB in this study had HIV RNA < 50 copies/ml at 24 months on HAART, which is similar to viral suppression in patients without TB (Figures 1 &2) and has been reported recently in another study [31]. In contrast, others have reported a high hazard ratio for virologic failure in patients with TB on ART [32].

Since many patients in Sub-Saharan Africa present to the health facilities with advanced disease stages and low CD4 cell counts [24], they may have limited advantage for CD4+ recovery after ART [17]. However, despite the overall lower baseline CD4+ count of the healthy Ethiopians [33], and the lower CD4+ count at ART start (197 cells/ μl ) in HIV patients without TB in this study, the increase in CD4+ cells after two years on ART (332 cells/μl) was comparable with a recent report from Ethiopia [34], and other reports from Africa [24], in low-income countries (Africa, Latin America and Asia) [35], and the United States [27,36]. The mean CD4+ increase by 24 month on ART in this study (6.7 cells/μl/month) was also comparable to a report from South Africa [37].

However, 45% of the non-TB patients in this study had immunologic failure (an increase of <50 CD4+ cells/μl) at 6 months, while 32% failed to restore CD4+ T cell count to ≥ 200 cells/μl by 2 years on ART, which is similar to a study from Nigeria [38]. Similar to Lifson *et al.*[36], advance WHO stages and lower CD4+ count at baseline were risk factors for immunologic failure, which strongly suggests the need for earlier identification of eligible patients and initiation of HAART.

Furthermore, since more than 50% of the HIV/TB patients in Sub-Saharan Africa presented to the health facilities at advanced disease stages and start ART at CD4+ counts of 100–150 cells/μl [16,24] the benefit of patients on ART could be limited [28,39]. More than 50% of the HIV/TB patients in our cohort had advanced disease stage at enrolment and the CD4+ count at ART initiation was 110 cells/μl (Table 2). Interestingly, however, we observed no difference in the CD4+ cell increase over time on HAART in patients with and without TB as reported by Lawn SD *et al.*[40] and Dronda F *et al.*[31]. In contrast, others reported reduced CD4+ recovery after ART in patients with TB [15,41,42]. Interestingly, although the study sample was small, we did observe reduced CD4+ recovery in HIV/TB patients co-infected with HBV (Figure 3A) as reported by Pe’ rez-Molina JA *et al.*[43].

Overall, the total CD4+ count achieved by 2 years on HAART in patients with TB (290 cells/μl ) and without TB (332 cells/μl) in this study was comparable to findings from African and other developed countries, although it was still lower compared to the healthy Ethiopian populations (754.8 cells/μl). This poses the question whether immune function is restored after 2 year of HAART. Therefore, the *Mtb* specific immune restoration after 18 month on HAART was investigated.

It has been estimated that, if HAART is accessible to all patients with CD4+ < 200 cells/μl and would restore optimal immune responses specific to *Mtb*, the cumulative incidence of TB would decrease by 22% over 20 years [44]. However, if immune restoration to *Mtb* is incomplete, there would be a substantial number of patients on HAART which are continuously at high risk for TB. In this study we measured the level of IFN-γ, a cytokine which plays a key role in the control of *Mtb* infection [45] in response to *Mtb* specific antigen (ESAT-6/CFP-10) [46].

We observed lower IFN-γ secretion in HIV negative TB patients compared to LTBI individuals at baseline as reported by Hanna LE *et al.*[47]. Coinfection with HIV severely decreased the secretion of IFN-γ in both groups. Furthermore, in support to previous reports [41,42,48], IFN-γ production after 18 month of HAART was not restored in HIV patients with TB while it was normalized in those with LTBI. Among the possible factors contributing to the impaired IFN-γ response in the HIV/TB patients are exhaustion of immune system [47] defined as a reduced proliferation of immune cells and impaired cytokine production due to infection with HIV [49] and *Mtb*[50] , and depletion of *Mtb* specific CD4+ cells due to direct infection with HIV [15]. Our observation that there was no correlation between CD4+ count recovery after HAART and level of IFN-γ production in the HIV + TB + patients, unlike to that of HIV-TB + patients where there was a strong correlation between CD4+ T cell recovery and IFN-γ production following TB treatment (Figure 5), suggests that other factors may play a role in the impaired functional recovery of *Mtb* specific immune responses in the HIV + TB + patients. Among the strategies proposed to boost immune restoration specific to TB after HAART are early initiation of HAART and isoniazid prophylaxis, and adjunctives such as BCG vaccination or co-administration of IL-2 [48,51].

Overall, addressing the long-term outcome of HAART by comprehensively measuring the key parameters of ART responses in a well defined cohort of patients with and without active TB and LTBI is the major strength of this study. The study addressed immune responses after HAART not only by measuring the absolute CD4+ cell recovery as a proxy for immune restoration but also by measuring IFN-γ response specific to *Mtb.* However, the fact that there is no golden standard definition of LTBI could be counted as limitations of the study

## Conclusions

In this observational cohort study, we showed sustained outcomes of long-term HAART in HIV patients with and without TB and LTBI as evidenced by clinical, immunologic and virologic data. Advanced pre-ART disease stages were the risk factors for diminished CD4+ and virologic responses to HAART and high mortality, which strongly indicated the need of early identification of eligible patients and early access to care and treatment. *Mtb* specific immune reconstitution in HIV/TB patients remained impaired after 18 months on HAART, which suggested the need of strong prevention, earlier diagnosis, and treatment of TB, as well as earlier initiation of HAART*.* Factors contributing to impaired *Mtb* specific immune restoration in HIV/TB patients after HAART need to be investigated in order to develop intervention methods which could boost the immune response. In addition, we should do further study on the immunological mechanisms associated with HIV/TB coinfection.

## Materials and methods

### Study populations and settings

This observational cohort study was performed from April 2007-February 2011 at St Peter Specialized Referral TB Hospital, Akaki and Kality Health centers in Addis Ababa, Ethiopia. Adults of both sexes who were naïve to ART and TB treatment were enrolled after informed and written consent was sought.

Diagnosis of active tuberculosis (TB) was based on both clinical and bacteriological evidences. At least two sputum smears stained by the Ziehil-Neelsen direct method were required to be microscopy positive for Acid Fast Bacilli (AFB) [52]. Except for TB patients, Mantoux Tuberculin Skin Testing (TST) for tuberculin was done for all participants. A diameter of skin induration with ≥ 10 mm in HIV un-infected, and ≥ 5 mm in HIV-infected individuals was graded as TST positive (TST+), and was considered as a LTBI [52].

The study participants were enrolled in 5 clinical groups: HIV patients with TB (HIV + TB+), and without TB (HIV + TB-) [sub-grouped further as Tuberculin Skin Test (TST) positive (HIV + TST+) and TST negative HIV + TST-], HIV negative TB cases (HIV-TB+), HIV-TST+, and controls (HIV-TST-). After enrolment, the HIV + TB+, HIV + TB-, and HIV-TB + groups were scheduled for follow-up (FU) clinic visits at sixth month (M6), M18 and M24.

At enrolment and during FU visit, each participant was interviewed using a standard questionnaire and detailed clinical, anthropometric and demographic data were recorded by a clinician or a nurse. A total of 20 ml heparinized venous blood was collected and transported immediately to the National HIV Referral Laboratory (NHL), at EHNRI. HAART was prescribed for eligible HIV patients and anti-TB treatment for all the TB cases free of charge as per the national guidelines. Antibiotic prophylaxis was also prescribed by the physician at enrolment or during the clinic follow-up visits [52].

### Laboratory tests

Laboratory examinations of blood were performed by automated machines following the manufacturer’s protocol. Hematological values were determined using Cell Dyn (Abbott laboratories, Abott Par IC Jl 60064, USA); CD4+ T cell counts were determined using Becton Dickinson (BD) FACSCalibur (Becton Dickinson, San Jose, USA); and Plasma HIV-1 RNA load was measured using the NucliSens EasyQ NASBA diagnostic 2007/1 (Organon, Teknika) which has a detection range of 50–3,000,000 copies/ml. The level of IFN-γ (pg/ml) in the 7th day whole blood culture supernatant stimulated with *Mtb* specific antigen (ESAT-6/CFP-10) was measured by xMAP multiplex technology (Luminex, Austin TX, USA), using Biosource reagents (Biosource, Camarillo, USA), and analyzed with the STarStation v2.0 software (Applied Cytometry Systems, United Kingdom) as described previously [53].

#### Definitions

Based on data from different studies [54,55], the national [56] and international [11] guidelines, and considering the small sample size in this study, we choose the following cut-off values to define HAART outcomes. *Body mass index (BMI)* (kg/m^2^) was categorized as malnutrition (BMI <18.50) and normal (18.50 ≥ BMI ≤ 24.99), and anemia was defined as hemoglobin (Hgb) <12 g/dl [57]; v*irologic success* was defined as achieving a viral suppression (HIV RNA < 50 copies/ml, or lower than the detection level (LDL) of the assay) after HAART, and *virologic failure* as a single HIV RNA >5000 copies/ml after a minimum of 6 months on HAART [11]. *Immunologic success* was defined as an increase of ≥ 50 CD4+ cells/μl at M6 and ≥100 cells/μl at M18 and M24 from that at ART initiation; *immunologic failure* as a failure to increase ≥ 50 cells/μl at M6, or ≥100 cells/μl at M18 and M24; and *super-responders* as patients able to achieve CD4+ count of >500 cells/μl after 2 years of HAART.

#### Statistical analysis

Data entered using Microsoft Access (DBse XI) was double-checked for discrepancies. All data analysis was done using Intercooled STATA version 11.0 (College Station, Texas, USA). Descriptive analyses including counts and frequencies for categorical variables, and mean (standard deviation, SD) or median (interquartile range, IQR) for continuous variables were computed. Results were compared using chi-square test and Fisher’s exact test for categorical variables and non-parametric tests (Wilcoxon signed rank test and Mann–Whitney U test) for continuous variables. Fixed (sex, groups) and time-updated variables (age, CD4+ count, HIV RNA, BMI, Hgb, TLC, co-infections) were included in the logistic regression analysis to identify risk factors for failed immunologic and virologic responses to HAART. A p-value of < *0.05* was considered as statistical significant.

#### Ethical approval

This study was ethically approved institutionally, by the Scientific and Ethics Review Office (SERO), EHNRI; and nationally by the National Health Research Ethics Review Committee (NHRERC), Ethiopian Ministry of Science and Technology Agency (ESTA).

## Competing interests

The authors declare that they have no competing interests.

## Authors’ contributions

DK was a lead author on planning, implementation of the study, data analysis, and writing of the draft, interim and final version of the manuscript; GG and YA participated in different laboratory tests; AA participated in counseling the study participants, filling study questionnaires and sample collection; DW, TE and DB – participated in providing advice and help during data analysis and also offered inputs and recommendations during the draft, interim and final version of the manuscript. All authors have seen and approved the final manuscript.
